# A Multifunctional Neutralizing Antibody‐Conjugated Nanoparticle Inhibits and Inactivates SARS‐CoV‐2

**DOI:** 10.1002/advs.202103240

**Published:** 2021-11-10

**Authors:** Xiaolei Cai, Min Chen, Aleksander Prominski, Yiliang Lin, Nicholas Ankenbruck, Jillian Rosenberg, Mindy Nguyen, Jiuyun Shi, Anastasia Tomatsidou, Glenn Randall, Dominique Missiakas, John Fung, Eugene B. Chang, Pablo Penaloza‐MacMaster, Bozhi Tian, Jun Huang

**Affiliations:** ^1^ Pritzker School of Molecular Engineering University of Chicago Chicago IL 60637 USA; ^2^ Department of Chemistry University of Chicago Chicago IL 60637 USA; ^3^ Committee on Cancer Biology University of Chicago Chicago IL 60637 USA; ^4^ Department of Microbiology Howard Taylor Ricketts Laboratory University of Chicago Chicago IL 60637 USA; ^5^ Department of Surgery University of Chicago Chicago IL 60637 USA; ^6^ Department of Medicine University of Chicago Chicago IL 60637 USA; ^7^ Department of Microbiology‐Immunology Northwestern University Chicago IL 60611 USA

**Keywords:** COVID‐19, multifunctional nanoparticle, photothermal therapy, SARS‐CoV‐2, virus inactivation

## Abstract

The outbreak of 2019 coronavirus disease (COVID‐19), caused by severe acute respiratory syndrome coronavirus 2 (SARS‐CoV‐2), has resulted in a global pandemic. Despite intensive research, the current treatment options show limited curative efficacies. Here the authors report a strategy incorporating neutralizing antibodies conjugated to the surface of a photothermal nanoparticle (NP) to capture and inactivate SARS‐CoV‐2. The NP is comprised of a semiconducting polymer core and a biocompatible polyethylene glycol surface decorated with high‐affinity neutralizing antibodies. The multifunctional NP efficiently captures SARS‐CoV‐2 pseudovirions and completely blocks viral infection to host cells in vitro through the surface neutralizing antibodies. In addition to virus capture and blocking function, the NP also possesses photothermal function to generate heat following irradiation for inactivation of virus. Importantly, the NPs described herein significantly outperform neutralizing antibodies at treating authentic SARS‐CoV‐2 infection in vivo. This multifunctional NP provides a flexible platform that can be readily adapted to other SARS‐CoV‐2 antibodies and extended to novel therapeutic proteins, thus it is expected to provide a broad range of protection against original SARS‐CoV‐2 and its variants.

## Graphical abstract

A multifunctional neutralizing antibody‐conjugated nanoparticle is developed to capture and inactivate SARS‐CoV‐2. It efficiently captures SARS‐CoV‐2 pseudovirions and completely blocks viral infection of host cells in vitro through the surface neutralizing antibodies. In addition, its photothermal function inactivates viruses upon light irradiation. More importantly, it treats authentic SARS‐CoV‐2 infection in vivo outperforming neutralizing antibodies.

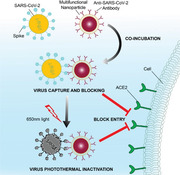

## 1 Introduction

Coronavirus disease 2019 (COVID‐19), resulting from severe acute respiratory syndrome coronavirus 2 (SARS‐CoV‐2) infection, has spread worldwide and caused a global pandemic.^[^ ^1^, ^2^ ^]^ Global epidemics initiated by emerging and reemerging viruses, including SARS‐CoV, H1N1, Zika virus, and Ebola virus have become increasingly prevalent over the past 80 years and are expected to increase in the future.^[^ ^3^ ^]^ However, the lack of an available treatment or therapeutic strategy remains a challenge to mount an effective protection against viral threats.

SARS‐CoV‐2 gains entry into cells through engagement of the receptor‐binding domain in the spike protein S1 subunit with the angiotensin‐converting enzyme 2 (ACE2) receptor on the host cell surface and subsequent viral fusion and entry mediated by the spike S2 subunit.^[^ ^4^, ^5^ ^]^ In response to viral infection, host macrophages and monocytes upregulate inflammatory cytokines often contributing to cytokine release syndrome.^[^ ^6^ ^]^ In addition, the virus can induce acute respiratory distress syndrome and cardiovascular damage, as well as increased mortality in patients.^[^ ^7^, ^8^, ^9^ ^]^

Vaccines to protect against SAR‐CoV‐2 infection have shown promising results in clinical trials and have been authorized for emergency use, but it may take months or years to vaccinate the world population due to the manufacturing and deployment processes.^[^ ^10^ ^]^ Moreover, it remains unclear whether vaccines will confer long‐term protection or be effective for newly emerging variants, further complicating the path toward ending the pandemic.^[^ ^11^ ^]^ Currently, there are limited therapeutic regimens proven to clear the viral infection in all patients evaluated.^[^ ^12^, ^13^, ^14^ ^]^ Transfusion of convalescent plasma from patients that have recovered from COVID‐19 into those with the active disease can reduce viral load and may limit the severity or duration of illness due to the presence of pre‐existing neutralizing antibodies specific for SARS‐CoV‐2.^[^ ^15^ ^]^ However, intravenous administration of convalescent plasma involves logistical hurdles, including availability of donor plasma and the need for a designated medical facility.^[^ ^16^ ^]^

In addition, antibody‐dependent enhancement (ADE), caused by binding of non‐neutralizing antibodies to virus, which has been observed in dengue virus,^[^ ^17^ ^]^ Zika virus,^[^ ^18^ ^]^ SARS‐CoV,^[^ ^19^ ^]^ and SARS‐CoV‐2,^[^ ^20^ ^]^ remains a concern for the development and application of SARS‐CoV‐2 antibody‐based vaccines, therapies, and convalescent plasma because it can further aggravate a patient's condition through enhancement of the viral infection.^[^ ^21^, ^22^, ^23^, ^24^ ^]^ High‐affinity monoclonal neutralizing antibodies against SARS‐CoV‐2 may circumvent some of the potential risk of ADE, as they often display much higher affinities for the SARS‐CoV‐2 spike protein (<1 nM)^[^ ^25^, ^26^, ^27^ ^]^ than for ACE2 (≈15–40 nM)^[^ ^28^, ^29^, ^30^, ^31^ ^]^ and can be produced in mass scale. Not surprisingly, several monoclonal neutralizing antibodies cloned using B cells from COVID‐19 patients have been recently shown to effectively block the interaction between SARS‐CoV‐2 spike protein and ACE2.^[^ ^32^, ^33^, ^34^, ^35^, ^36^ ^]^ However, previous studies have reported that soluble antibodies are mostly eliminated from lungs within 24 h^[^ ^37^, ^38^, ^39^ ^]^ through mucociliary clearance^[^ ^40^ ^]^ or alveolar macrophages^[^ ^41^ ^]^ after pulmonary administration. As a result, the rapid clearance and short lung retention time of soluble antibodies may limit the effectiveness of antibody therapy, especially for local delivery.^[^ ^38^, ^42^ ^]^ Furthermore, the emergence of multiple variants of SARS‐CoV‐2, which may lead to more severe symptoms, brings more concerns on the efficacy of current therapies and vaccines.^[^ ^43^, ^44^ ^]^

**Scheme 1:**
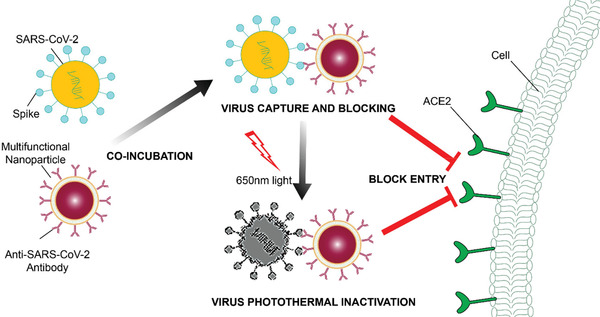
Schematic illustration of the multifunctional NPs for the capture (by antibody) and inactivation (by photothermal) of SARS‐CoV‐2.

Nanomaterial‐based approaches offer promising alternatives to using antibodies alone for virus detection,^[^ ^45^, ^46^, ^47^, ^48^ ^]^ vaccine delivery,^[^ ^49^, ^50^ ^]^ and viral capture.^[^ ^51^, ^52^, ^53^, ^54^ ^]^ For example, engineered liposomes,^[^ ^55^ ^]^ nanosponges,^[^ ^56^ ^]^ and exosomes^[^ ^57^ ^]^ have been utilized to target other viruses in addition to SARS‐CoV‐2. However, these methods are only capable of capturing the virus; an effective approach to inactivate the virus—which may prevent ADE and be effective for different variants—remains to be seen. Photothermal nanoparticles (NPs) capable of eliminating tumors and microorganisms have been developed and have led to potent killing of the targets without damaging the surrounding healthy tissues.^[^ ^58^, ^59^, ^60^ ^]^ While there are a few studies applying inorganic photothermal NPs for virus inactivation,^[^ ^61^, ^62^ ^]^ organic photothermal NPs, which possess better biocompatibility than inorganic photothermal NPs, are lacking.^[^ ^63^ ^]^ Herein we report the development of a strategy utilizing multifunctional organic photothermal NPs decorated with high‐affinity neutralizing antibodies designed to effectively capture and inactivate SARS‐CoV‐2 (**Scheme** **1**). Each multifunctional NP contains an amphiphilic polymer shell encapsulating a semiconducting polymer core capable of generating intense local heat after being excited by suitable light sources. The NP surface is functionalized with a high affinity (0.07 nM) monoclonal neutralizing antibody specific to the SARS‐CoV‐2 spike protein, which possesses excellent SARS‐CoV‐2 neutralizing efficiency. The resultant multifunctional NPs can selectively and efficiently capture and block SARS‐CoV‐2, completely preventing the entry of SARS‐CoV‐2 into host cells—an improvement over antibody treatment alone. Upon excitation by a 650‐nm light‐emitting diode (LED) which possesses a more desirable safety profile compared to conventional laser excitation,^[^ ^64^, ^65^ ^]^ the multifunctional NPs can further inactivate the virus by the auxiliary photothermal function. Furthermore, the multifunctional NPs possess advantageous properties for lung delivery and retention, which can overcome the limitation of rapid clearance of antibodies in the lung.^[^ ^38^, ^42^ ^]^ The unique design of our multifunctional NPs not only enables antibody‐mediated neutralizing function in capturing SARS‐CoV‐2, but may also provide a strategy to mitigate the potential risks of ADE and new, more infectious SARS‐CoV‐2 variants through direct heat inactivation of the virus. In addition, as the first organic photothermal NP for SARS‐CoV‐2 inactivation, our NP can also serve as a proof‐of‐concept platform for future viral therapies.

## 2 Results and Discussion

**Figure 1:**
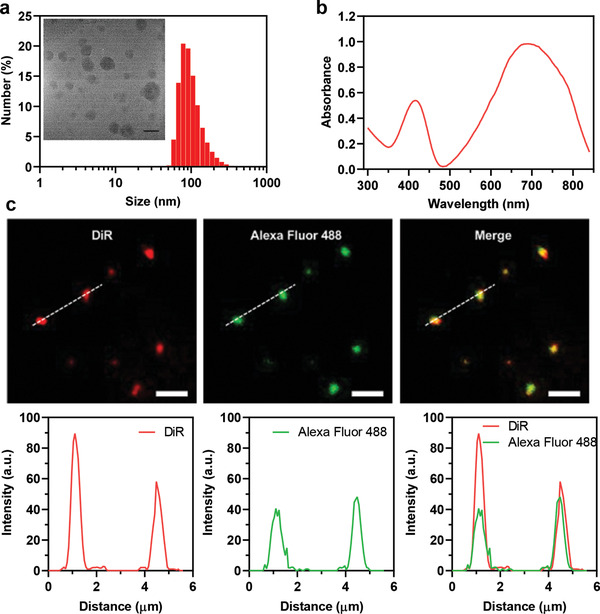
Characterization of the multifunctional NPs. a) DLS size distribution of the multifunctional NPs. Inset: TEM image of the NPs. Scale bar: 50 nm. b) UV–vis absorption spectrum of the multifunctional NPs. c) Fluorescent images and fluorescence intensity profiles of the neutralizing antibody‐conjugated multifunctional NPs labeled by DiR (red) and immunostaining by a secondary Alexa Fluor‐488 anti‐IgG2b antibody (green) and overlay. Line scan is used to indicate the fluorescence profile and co‐localization for single NPs. Excitation: 488 nm (green channel) and 740 nm (red channel). Scale bars: 2.5 µm.

The multifunctional NPs were prepared by using 1,2‐distearoyl‐sn‐glycero‐3‐phosphoethanolamine‐*N*‐[carboxy(polyethylene glycol)‐2000, NHS ester] (DSPE‐PEG_2000_‐NHS) as the matrix to encapsulate poly[2,6‐(4,4‐bis‐(2‐ethylhexyl)‐4H‐cyclopenta [2,1‐b;3,4‐b′]dithiophene)‐alt‐4,7(2,1,3‐benzothiadiazole)] (PCPDTBT) through self‐assembly.^[^ ^66^ ^]^ Subsequently, an anti‐SARS‐CoV‐2 neutralizing antibody (IgG2b) confirmed to neutralize SARS‐CoV‐2 via bioluminescence‐based pseudovirus neutralization assay (Figure S1, Supporting Information) was conjugated to the NPs. In order to ensure covalent attachment of antibodies to the NP surface, we utilized the commonly employed NHS ester coupling, as there exist multiple lysine residues on an antibody.^[^ ^67^ ^]^ Using dynamic light scattering (DLS), we determined the hydrodynamic diameters of the NPs to be ≈70 nm, which was validated by transmission electron microscopy (TEM) (**Figure** **1**a). In addition, the NPs exhibited good stability without forming any aggregation or precipitation after being stored in aqueous dispersions at 4 °C for several weeks (Figure S2, Supporting Information). The NPs displayed excellent absorption from 500 to 850 nm in the red and near‐infrared region (Figure 1b), favoring the use of infrared excitation to enable deep tissue penetration^[^ ^66^ ^]^ as well as photothermal‐mediated inactivation of SARS‐CoV‐2.

Surface conjugation of the anti‐SARS‐CoV‐2 neutralizing antibody was further validated by fluorescent imaging analysis. Briefly, DiOC_18_(7) (DiR) lipophilic dye capable of emitting a fluorescent signal at ~780 nm was co‐encapsulated into the photothermal NPs (Figure 1c). After immunostaining with a secondary Alexa Fluor‐488‐labeled anti‐IgG2b antibody, subsequent visualization by fluorescence microscopy demonstrated good colocalization between the Alexa Fluor‐488 signal and the DiR signal (Pearson's correlation coefficient ≈ 0.9, calculated by ImageJ), confirming conjugation of the anti‐SARS‐CoV‐2 antibody on the NP surface. We quantified the antibody conjugation efficiency by monitoring the fluorescent signal of the NPs and antibodies via flow cytometry^[^ ^68^ ^]^ and determined that ~22% of antibodies were successfully coated onto the NP surface (Figure S3, Supporting Information) with random conjugation sites and antibody orientation.^[^ ^69^ ^]^

**Figure 2:**
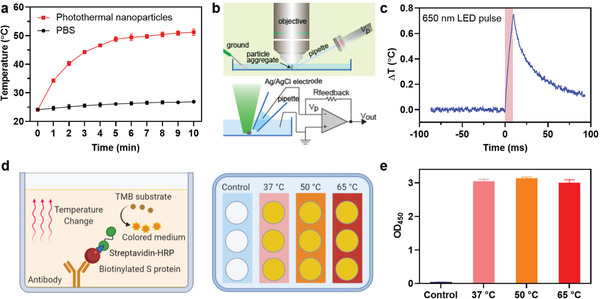
Photothermal characterization of the multifunctional NPs. a) Temperature changes of the multifunctional NPs (200 µL) and 1 × PBS (200 µL) after excitation with a 650‐nm LED at the indicated times. Error bars indicate ± SEM. *n* = 3 per group. b) Schematic diagram (top) and electrical diagram (bottom) of voltage clamp microscope setup used to measure local temperature change. c) A representative trace of temperature increase of the multifunctional NPs during 10 ms excitation with a 650‐nm LED at 1.7 W cm^−2^ intensity. *τ*: the elapsed time required for the temperature response to decay to 1/*e* of initial value. d) Schematic representation of ELISA for testing the interaction between anti‐SARS‐CoV‐2 neutralizing antibody and spike protein at different temperatures. e) Optical density (OD_450_) of ELISA samples at different temperatures. Control: no antibody is added (37 °C). Error bars indicate ± SEM. *n* = 3 per group.

Since the multifunctional NPs exhibited excellent absorption in the near‐infrared region, we hypothesized that they could effectively generate enough local heat for SARS‐CoV‐2 inactivation after light excitation with a suitable wavelength, as previous studies revealed that heat can inactivate coronavirus pathogens.^[^ ^70^ ^]^ To test this hypothesis, the NPs were dispersed in phosphate‐buffered saline (1 × PBS; pH 7.4) at a concentration of 100 µg mL^−1^, then subjected to 650‐nm LED excitation with a power density of 250 mW cm^−2^ which would be suitable to safely excite healthy cells and tissues.^[^ ^64^, ^65^ ^]^ As expected, a time‐dependent temperature increase from 22 to 50 °C was observed within 10 min after light exposure (**Figure** **2**a). In contrast, a temperature change of less than 2 °C was observed in PBS alone, indicating the effect was specific to the photothermal NPs (Figure 2a). Since the global temperature of the entire solution exceeded 50 °C, we rationalized that the local temperature near the multifunctional NP surface would be higher, as was previously reported.^[^ ^71^ ^]^ To test this hypothesis, we further evaluated local temperature changes following excitation using an LED‐coupled voltage‐clamp setup (Figure 2b) to determine the transient photothermal response of the NPs.^[^ ^72^ ^]^ The NPs were drop‐cast onto a glass coverslip and the aggregate was covered with a PBS solution. The glass micropipette electrode was placed in close proximity to the surface of the aggregated NPs. Then, a 650‐nm LED was used to deliver 10 ms light pulses and the current response through the pipette was measured (Figure S4, Supporting Information). The analysis revealed a 0.7 °C increase in the local temperature after each light pulse (Figure 2c), which corresponds to an initial heating rate of 70 °C s^−1^. After the end of the pulse, the temperature decayed with a ≈28 ms time constant (*τ*, Figure 2c).^[^ ^73^ ^]^ While the local temperature change observed in the measurement is lower than observed previously for silicon nanostructures,^[^ ^74^, ^75^ ^]^ the light density used for excitation is more than 100‐times lower due to application of LED rather than focused laser. Additionally, the longer decay time constant suggests higher thermal resistance at the organic NP‐water interface compared to inorganic nanostructures, resulting in slow heat dissipation and higher local temperature.^[^ ^72^ ^]^ The measurements further suggest a strong photothermal response produced by the NPs and low heat dissipation to aqueous solution. We next investigated the effect of temperature variation on antibody binding affinity to spike protein by ELISA (Figure 2d). After performing the ELISA and heating the samples to 37, 50, or 65 °C on a hotplate, no significant difference in the binding between anti‐SARS‐CoV‐2 neutralizing antibody and spike protein was observed (Figure 2e), which is consistent with a previous study.^[^ ^76^ ^]^ The data suggest that the temperature rise induced by LED treatment will have a negligible effect on the SARS‐CoV‐2 sequestration effect by the multifunctional NPs. Taken together, these data suggest that our NPs can potentially capture and block SARS‐CoV‐2 through surface neutralizing antibodies and inactivating the virus through photothermal effect.

**Figure 3:**
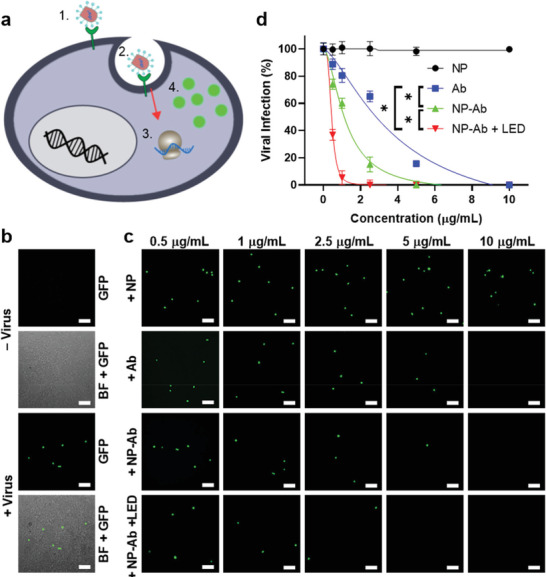
SARS‐CoV‐2 VSV‐GFP pseudovirus infection in ACE2/HEK293T cells. a) Schematic illustration of SARS‐CoV‐2 VSV‐GFP pseudovirus infecting ACE2/HEK293T cells: the spike protein on the pseudotyped virus surface binds to ACE2 (1) and infects the cell (2), releasing its RNA to be transiently expressed by the host (3) into GFP (4). b) Representative fluorescent and brightfield (BF)‐GFP merge images of ACE2/HEK293T cells before and after incubation with SARS‐CoV‐2 VSV‐GFP. Scale bars: 100 µm. c) Representative fluorescent images of ACE2/HEK293T cells after incubation with SARS‐CoV‐2 VSV‐GFP treated by different concentrations of the non‐modified NPs (NP), neutralizing antibody (Ab), neutralizing antibody‐conjugated multifunctional NPs (NP‐Ab)， or NP‐Ab + 650‐nm LED excitation at 250 mW cm^−2^ for 10 min (NP‐Ab + LED). Scale bars: 100 µm. d) Quantification of SARS‐CoV‐2 VSV‐GFP infectivity after treatment with different concentrations of NP, Ab, NP‐Ab, or NP‐Ab + LED. IC_50_ ≈ 3.893 (Ab), 1.189 (NP‐Ab), and 0.442 (NP‐Ab + LED) µg mL^−1^. Error bars indicate ± SEM. *n* = 3 per group. Statistical significance is determined by sum‐of‐squares F test; * *P* < 0.05.

Next, we sought to determine whether we could use the newly engineered multifunctional NPs to inhibit infection of ACE2‐expressing host cells by SARS‐CoV‐2. Due to the high risk of infection associated with authentic SARS‐CoV‐2, we utilized replication‐deficient viruses pseudotyped with the SARS‐CoV‐2 spike protein in order to evaluate the efficacy of the NPs. Pseudotyped viruses have been routinely employed to provide significantly safer conditions in which to study highly infectious viruses.^[^ ^77^, ^78^, ^79^, ^80^ ^]^ As an initial proof‐of‐concept, we utilized vesicular stomatitis virus pseudotyped with the SARS‐CoV‐2 spike protein (SARS‐CoV‐2 VSV‐GFP). The SARS‐CoV‐2 VSV‐GFP pseudovirus enables transient expression of green fluorescent protein (GFP) upon entry into the host cell (**Figure** **3**a), facilitating direct monitoring of viral uptake following incubation with the NPs and photothermal treatment. In the absence of NPs, HEK293T cells engineered to overexpress ACE2 (ACE2/HEK293T) were susceptible to pseudovirus infection as demonstrated by GFP expression after 24 h incubation (Figure 3b). To monitor the effect of the multifunctional NPs on virus entry, ACE2/HEK293T cells were simultaneously infected with SARS‐CoV‐2 VSV‐GFP preincubated with a dilution series of non‐modified NPs, neutralizing antibody (Ab), or neutralizing antibody‐conjugated multifunctional NPs (NP‐Ab) with or without LED pretreatment, then visualized by fluorescence microscopy. The viral inactivation efficiencies were determined by quantifying fluorescent cells and normalizing the values at each concentration to that of the cells infected with pseudovirus only. The actual viral inactivation efficiencies of the NP‐Ab were further calculated using the effective NP‐Ab concentrations corrected to account for the 22% antibody conjugation efficiency to eliminate the effect of free antibodies existing in the NP solutions (See detailed calculations in the Supporting Information). No change in viral infection was observed following treatment with non‐modified NPs. To validate the necessity of neutralizing antibodies on the surface of the NPs for trapping and inactivating the virus, we applied LED excitation to SARS‐CoV‐2 VSV‐GFP incubated with the non‐modified NP. As expected, the non‐modified NP elicited a negligible effect on viral infection (Figures S6, Supporting Information), indicating the importance of surface neutralizing antibody conjugation. In contrast, a dose‐dependent (0.5–10 µg mL^−1^) containment of the pseudovirus was observed upon addition of the Ab or NP‐Ab, showing IC_50_ values of 3.893 and 1.189 µg mL^−1^, respectively, representing a ~3‐fold improvement in inhibition (Figures 3c,d). While the NP‐Ab can completely inhibit the viral infection, the 650‐nm LED excitation (250 mW cm^−2^, 10 min) further improved the inhibition efficiency of viral infection with an IC_50_ value of 0.442 µg mL^−1^, demonstrating a ~9‐fold improvement over soluble Ab and a ~3‐fold enhancement compared to the NP‐Ab in the absence of excitation (Figures 3c,d). Due to the 22% conjugation efficiency to the NP surface, we further explored whether immobilization of the neutralizing antibody provides a benefit compared to treatment with the soluble form. Simultaneous infection of ACE2/HEK293T cells with SARS‐CoV‐2 VSV‐GFP preincubated with a dilution series of non‐modified NP and soluble Ab (NP + Ab) with or without LED excitation led to significantly lower viral inactivation efficiency as compared to the NP‐Ab (Figure S7, Supporting Information), confirming the importance of antibody conjugation to the NPs.

**Figure 4:**
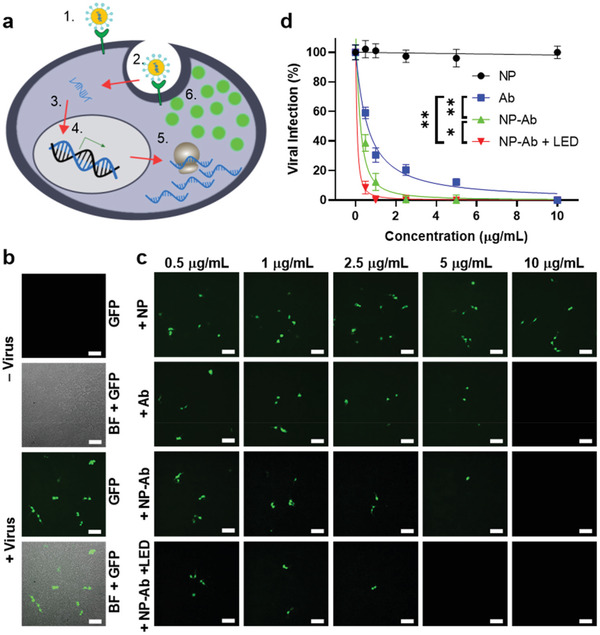
SARS‐CoV‐2 lentivirus‐GFP pseudovirus infection in ACE2/HEK293T cells. a) Schematic illustration of SARS‐CoV‐2 lentivirus‐GFP pseudovirus infecting ACE2/HEK293T cells. The spike protein on the pseudotyped virus surface binds to ACE2 (1) and infects the cell (2). The viral RNA is integrated into the host DNA (3) where it constitutively transcribes (4) and translates it (5) into GFP (6). b) Representative fluorescent and BF‐GFP merge images of ACE2/HEK293T cells before and after incubation with SARS‐CoV‐2 lentivirus‐GFP. Scale bars: 100 µm. c) Representative fluorescent images of ACE2/HEK293T cells after incubation with SARS‐CoV‐2 lentivirus‐GFP treated by different concentrations of NP, Ab, NP‐Ab, or NP‐Ab + LED. Scale bars: 100 µm. d) Quantification of SARS‐CoV‐2 lentivirus‐GFP infectivity after treatment with different concentrations of NP, Ab, NP‐Ab, or NP‐Ab + LED. IC_50_ ≈ 0.593 (Ab), 0.213 (NP‐Ab), and 0.076 (NP‐Ab + LED) µg mL^−1^. Error bars indicate ± SEM. *n* = 3 per group. Statistical significance is determined by sum‐of‐squares F test; * *P* < 0.05; ** *P* < 0.01.

To confirm this effect was not dependent on the structure of the pseudovirus, we utilized a second replication‐deficient lentivirus pseudotyped with SARS‐CoV‐2 spike protein (SARS‐CoV‐2 lentivirus‐GFP) which stably integrates a gene encoding a GFP reporter into the host genome after infection (**Figure** **4**a). Viral inactivation efficiencies were determined using the same method described above (See detailed calculations in the Supporting Information). As expected, in the absence of Ab or NP‐Ab or treatment with non‐modified NP, the pseudotyped lentivirus infected ACE2/HEK293T cells, as indicated by GFP expression monitored at 48 h post‐infection (Figure 4b,c). Moreover, exposure of SARS‐CoV‐2 lentivirus‐GFP treated with non‐modified NP to LED excitation had a negligible effect on preventing viral infection (Figure S6, Supporting Information). However, after preincubation with the Ab or NP‐Ab, the SARS‐CoV‐2 lentivirus‐GFP infection was inhibited in a dose‐dependent fashion with increasing antibody concentrations (Figure 4c) corresponding to IC_50_ values of 0.593 and 0.213 µg mL^−1^, respectively, displaying a similar ~ 3‐fold improvement to that observed with the SARS‐CoV‐2 VSV‐GFP pseudovirus (Figure 4d). An improved inhibition efficiency was observed, showing an IC_50_ value of 0.076 µg mL^−1^ after pretreatment of the NP‐Ab and pseudovirus with 650‐nm LED excitation (250 mW cm^−2^, 10 min), demonstrating an ~8‐fold improvement over soluble Ab and a ~3‐fold enhancement compared to the NP‐Ab in the absence of excitation. The results are consistent with the enhancement demonstrated with the SARS‐CoV‐2 VSV‐GFP pseudovirus (comparing Figure 3 with Figure 4). The mixture of non‐modified NP and soluble Ab (NP + Ab) with or without LED excitation also shows much lower viral inactivation efficiency than that of the NP‐Ab (Figure S8, Supporting Information). Taken together, these results suggest that the NP‐Ab inhibit viral infection in a spike protein‐dependent mechanism, regardless of pseudovirus structure. Moreover, the NP‐Ab provides an enhanced virus capture ability compared with the soluble Ab and can further improve the inhibition of viral infection via photothermal‐mediated inactivation.

**Figure 5:**
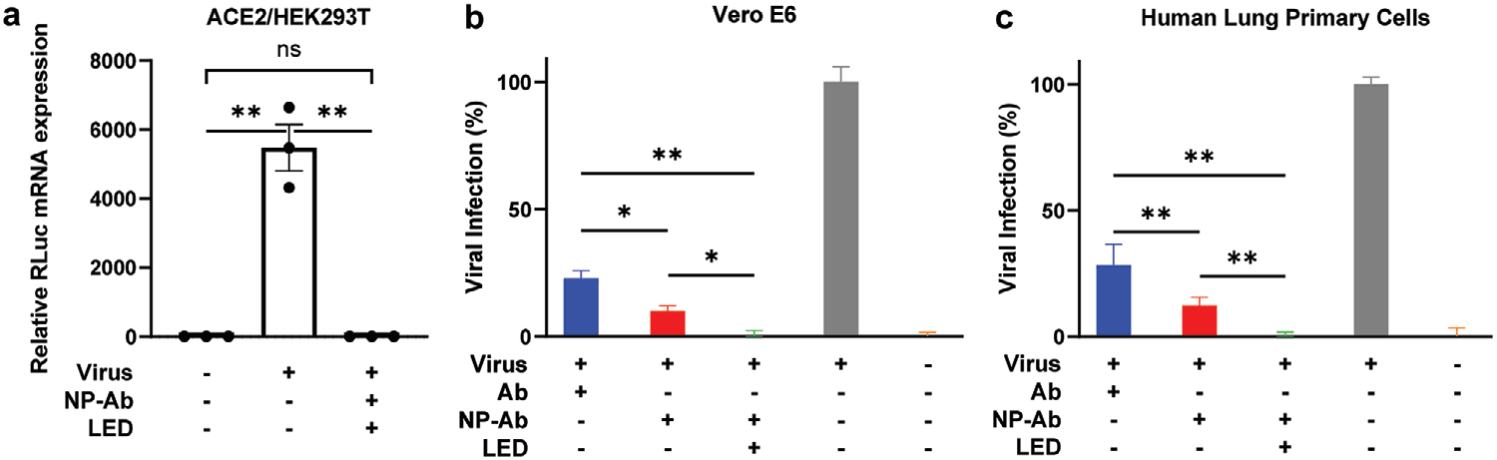
SARS‐CoV‐2 lentivirus‐luciferase pseudovirus inactivation. a) qRT‐PCR quantification of luciferase mRNA expression in ACE2/HEK293T cells after incubation with SARS‐CoV‐2 lentivirus‐luciferase treated by the NP‐Ab and 650‐nm LED. Error bars indicate ± SEM. *n* = 3 per group. Statistical significance is determined by two‐tailed unpaired *t*‐test; ** *P* < 0.01; ns: not significant, *P* > 0.05. b,c) SARS‐CoV‐2 lentivirus‐luciferase infectivity to Vero E6 cells (b) and human lung primary cells (c). Infectivity scores of different treatments are calculated based on the luciferase signals by normalizing to virus alone. Error bars indicate ± SEM. *n* = 5 per group. Statistical significance is determined by two‐tailed unpaired *t*‐test; * *P* < 0.05; ** *P* < 0.01.

In order to further quantify multifunctional NP‐mediated inhibition, we utilized a SARS‐CoV‐2‐pseudotyped lentivirus bearing a luciferase reporter gene (SARS‐CoV‐2 lentivirus‐luciferase) and monitored viral infection via real‐time quantitative PCR (qRT‐PCR). SARS‐CoV‐2 lentivirus‐luciferase was pre‐incubated with NP‐Ab and treated by LED excitation, then subsequently added to ACE2/HEK293T cells. We chose to treat the SARS‐CoV‐2 lentivirus‐luciferase with a single concentration (5 µg mL^−1^) to test whether such a concentration can achieve complete inhibition of viral infection based on the dose response curves shown in Figure 4. As expected, after 48 h no luciferase expression was observed with NP‐Ab and LED treatment indicating efficient virus inactivation (**Figure** **5**a), consistent with the fluorescent microscopy data shown in Figure 4.

To assess the inhibition efficiency of the multifunctional NPs in the presence of endogenously expressed ACE2, we evaluated function of the multifunctional NPs using African monkey kidney epithelial Vero E6 cell line^[^ ^81^ ^]^ and human lung primary cells^[^ ^54^, ^82^ ^]^ infected with SARS‐CoV‐2 lentivirus‐luciferase. As shown in Figures 5b,c, the NP‐Ab exhibits better inhibition of viral infection (~90% inhibition) than the soluble Ab alone (~70% inhibition), while the NP‐Ab + LED completely blocked viral infection, highlighting the enhancement by the photothermal effect inherent to the multifunctional NPs. These results were consistent with those obtained in ACE2/HEK293T cells (Figures 3 and 4). Taken together, these data demonstrate the excellent performance of our multifunctional NPs in blocking and inactivating SARS‐CoV‐2 pseudoviruses and suggest they may be amenable to inhibiting infection by the authentic virus.

**Figure 6:**
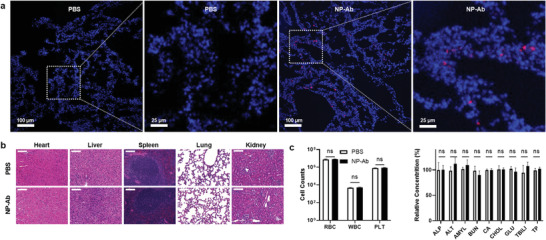
In vivo studies of the distribution and biosafety of the multifunctional NPs. a) Fluorescent images of mouse lung cryosections at day 3 post intratracheal injection of PBS or the multifunctional NPs. b) H&E staining of sections from different organs at day 3 post intratracheal injection of PBS or the multifunctional NPs. Scale bars: 50 µm. c) Blood analysis at day 3 post intratracheal injection of PBS or the multifunctional NPs. Error bars indicate ± SEM. *n* = 5 per group. Statistical significance is determined by two‐tailed unpaired *t*‐test; ns: not significant, *P* > 0.05.

In order to investigate the biosafety profile of the multifunctional NPs for therapeutical purpose, we first evaluated in vitro cytotoxicity in different cells. No toxicity was observed after treating ACE2/HEK293T cells, human lung epithelial cell line A549, and human lung primary cells endogenously expressing ACE2 with a dilution series of the multifunctional NPs (Figures S9 and S10, Supporting Information). We next monitored distribution and toxicity in vivo by intratracheally injecting the multifunctional NPs into immunocompetent mice.^[^ ^52^ ^]^ At day 3 post‐injection, the mice were sacrificed to collect the organs and blood for histological analysis. Fluorescent images of lung cryosections revealed that the NPs were successfully delivered into the mouse lungs (**Figure** **6**a). As compared to soluble Ab, which is cleared from the lung within 24 h,^[^ ^38^, ^42^ ^]^ the multifunctional NPs have a longer retention time that could prolong the local therapeutic effect inside the lung. Hematoxylin and eosin (H&E) staining of lung, heart, liver, spleen, and kidney (Figure 6b) and blood analysis including blood cell counting and blood chemistry analysis (Figure 6c) revealed that there were no histological differences between the NP‐treated and PBS‐treated mice. These biosafety data provide support for the potential use of the lung‐delivered multifunctional NPs to treat SARS‐CoV‐2 in vivo.

**Figure 7:**
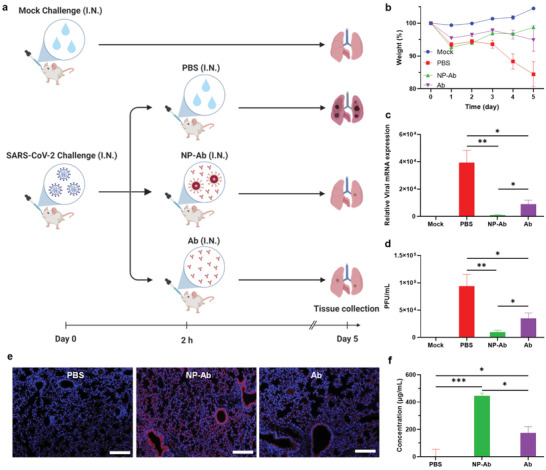
In vivo studies of the multifunctional NPs for SARS‐CoV‐2 treatment. a) Schematic illustration of the in vivo studies of authentic SARS‐CoV‐2 treatments by NP‐Ab or Ab. b) Mouse body weights after SARS‐CoV‐2 challenge under different treatments. Error bars indicate ± SEM. *n* = 5 per group. c) Quantification of SARS‐CoV‐2 mRNA expression in mouse lungs via qRT‐PCR at day 5 post SARS‐CoV‐2 challenge. Error bars indicate ± SEM. *n* = 5 per group. Statistical significance is determined by two‐tailed unpaired *t*‐test; * *P* < 0.05; ** *P* < 0.01. d) Viral titers in mouse lungs at day 5 post SARS‐CoV‐2 challenge. Error bars indicate ± SEM. *n* = 5 per group. Statistical significance is determined by two‐tailed unpaired *t*‐test; * *P* < 0.05; ** *P* < 0.01. e) Immunostaining of mouse lung sections after intranasal injection of PBS, NP‐Ab, or Ab. Blue channel: DAPI. Red channel: anti‐IgG‐PE. Scale bars: 50 µm. f) Concentrations of Ab in mouse lungs after intranasal injection of PBS, NP‐Ab, or Ab measured by ELISA. Error bars indicate ± SEM. *n* = 5 per group. Statistical significance is determined by two‐tailed unpaired *t*‐test; * *P* < 0.05; *** *P* < 0.001.

Finally, we evaluated the in vivo authentic SARS‐CoV‐2 containment efficacy of the multifunctional NPs by challenging K18‐hACE2 transgenic mice expressing human ACE2 with authentic SARS‐CoV‐2 (USA‐WA1/2020) (**Figure** **7**a). At 2 h post‐SARS‐CoV‐2 challenge, the NP‐Ab or soluble Ab were administrated intranasally into the mice for treatment. It was observed that the mice without any treatment show obvious weight loss after the SARS‐CoV‐2 challenge, while the mice treated by Ab or NP‐Ab show weight increase after the initial weight loss at day 1 (Figure 7b). The body condition scoring of mice also indicated a severe effect of the SARS‐CoV‐2 in the untreated group, while the Ab or NP‐Ab treatments can alleviate the effect (Figure S11, Supporting Information). After sacrificing the mice at day 5 post SARS‐CoV‐2 challenge, we monitored SARS‐CoV‐2 mRNA in mouse lungs by qRT‐PCR and determined that while soluble Ab led to a moderate reduction in viral RNA levels, NP‐Ab treatment contributed to a near‐complete inhibition, suggesting the multifunctional NPs were capable of inhibiting viral replication (Figure 7c). We further evaluated viral infection via plaque assay to quantify the effect of treatment on infectious SARS‐CoV‐2 viral particles. Gratifyingly, lung viral titers revealed that the NP‐Ab tremendously reduced the viral load in the lung, while the soluble Ab showed only moderate therapeutic effects against the virus, consistent with qRT‐PCR results (Figure 7d). The level of Ab in the mouse lung after NP‐Ab treatment or soluble Ab treatment was also investigated by immunostaining the lung tissue sections with anti‐IgG‐PE and DAPI (Figures 7e and Figure S12, Supporting Information), and ELISA quantification of the Ab in the lung homogenates (Figure 7f). Both samples showed that the Ab level in the mouse lung after NP‐Ab treatment is significantly higher than that soluble Ab treated group, which further supports the advantage of NP‐Ab in improving lung targeting and prolonging lung retention. Taken together, these observations are consistent with the in vitro results obtained using pseudoviruses and indicate that the multifunctional NPs possess excellent therapeutic effect against the authentic SARS‐CoV‐2 in vivo. More importantly, the multifunctional NPs also significantly outperform the soluble Ab possibly due to their higher avidity,^[^ ^54^ ^]^ better lung retention, as well as higher virus inactivation efficiency. Considering the fact that the NPs generate an excellent therapeutic effect after a single dose and can be easily fabricated into nasal sprays,^[^ ^83^ ^]^ they may be advantageous for repeated administration which could further improve their therapeutic effect. All these results indicate that the multifunctional NPs have the potential to serve as a feasible solution and an effective therapy for clinical SARS‐CoV‐2 treatment and generate an improved outcome compared to the current therapeutics, such as the monoclonal neutralizing antibody. Although the in vivo photothermal treatment was not performed due to limitations of our biosafety level 3 facilities and the availability of an appropriate light source, the in vitro experiments suggest it may further improve the therapeutic effect of the multifunctional NPs, which is also supported by previous studies. It has been reported that phototherapy can treat lung inflammation^[^ ^84^ ^]^ and lung injury^[^ ^85^ ^]^ in vivo after red light irradiation on the respiratory tract. In vivo ablation of lung cancer^[^ ^86^ ^]^ and other different types of cancers including skin cancer,^[^ ^87^ ^]^ breast cancer,^[^ ^88^ ^]^ glioblastoma,^[^ ^66^ ^]^ mammary cancer,^[^ ^89^ ^]^ and colorectal cancer^[^ ^90^ ^]^ were achieved by photothermal therapies using red/near‐infrared light excitation. In addition, in vivo photothermal killing of bacteria was realized.^[^ ^91^, ^92^ ^]^ A recent study also demonstrated successful photothermal treatment of neurotropic virus infection in the mouse brain.^[^ ^61^ ^]^ These studies demonstrate the feasibility of applying the multifunctional NPs with red/near‐infrared light excitation for in vivo photothermal inactivation of SARS‐CoV‐2. As the multifunctional NPs have a broad absorption spectrum in red/near‐infrared range, suitable light sources, LED or lasers, can be selected for in vivo studies to tune the excitation wavelength and power density for good penetration depth and photothermal effect. Meanwhile, the light can be delivered to the lung externally by light irradiation on the respiratory tract or internally by a flexible bronchoscopy,^[^ ^93^, ^94^ ^]^ which can further improve the light penetration and therapeutic effect of the multifunctional NPs. Future in vivo studies are needed to confirm these benefits.

## 3 Conclusion

In summary, we developed an antibody‐conjugated multifunctional NP for the capture and inactivation of SARS‐CoV‐2. The multifunctional NPs enabled successful capture of two different types of SARS‐CoV‐2 pseudoviruses in three different cell systems in vitro, outperforming the soluble neutralizing antibodies. In addition, the NPs displayed excellent photothermal effect to further inactivate the virus. The multifunctional NPs also exhibited excellent biosafety in vitro and in vivo and satisfactory lung delivery in mice. Most importantly, in vivo treatment with the multifunctional NPs in the presence of authentic SARS‐CoV‐2 was achieved showing a significantly improved therapeutic effect compared to soluble neutralizing antibodies and demonstrating their great potential for clinical SARS‐CoV‐2 treatment. The multifunctional NP provides a flexible platform that can be readily adapted to target other variants of SARS‐CoV‐2 via conjugation to different antibodies or other novel therapeutic proteins and can also be extended to other viruses, bacteria,^[^ ^60^ ^]^ and malignant cells.^[^ ^95^ ^]^ Furthermore, the photothermal function of the multifunctional NP could potentially serve to prevent ADE that may arise due to incomplete neutralization of the SARS‐CoV‐2 during SARS‐CoV‐2 infection and vaccination and actively inactivate different variants of SARS‐CoV‐2. In addition to the photothermal inactivation of virus, the red‐light treatment (650‐nm LED) applied in our study could potentially bring more benefits by modulating the immune system and reducing inflammation. Previous studies have reported that phototherapy using red light and near‐infrared light can reduce pulmonary inflammation and lung fibrosis in mice by downregulating pro‐inflammatory cytokines, upregulating the secretion of IL‐10 from fibroblasts and pneumocytes, and reducing collagen deposits in the lungs.^[^ ^96^, ^97^, ^98^ ^]^ Since pulmonary inflammation and lung fibrosis are commonly observed complications in critical patients with SARS‐CoV‐2 infection, the 650‐nm LED treatment in our study could potentially relieve these life‐threatening complications. Together with the efficient virus inactivation function, the excellent therapeutic effect of our multifunctional NPs can be further improved. Future studies will be conducted by using site‐specific conjugation methods such as site‐selective click chemistry methods^[^ ^69^ ^]^ to increase the surface antibody conjugation efficiency, control the antibody conjugation site and orientation, and purify the multifunctional NPs to further improve their therapeutic effect.

## Conflict of Interest

The authors declare no conflict of interest.

## Author Contributions

X.C. and J.H. conceived the ideas and designed the project. J.H. supervised the project. X.C. synthesized, characterized the nanoparticles, and conducted the in vitro viral infection experiments. X.C and M.C. conducted the flow cytometry experiment. A.P. measured the transient photothermal response and analyzed the data. Y.L. and J.S. performed the electron microscopy experiment and analyzed the data. N.A. and J.R. contributed to the imaging data analysis. M.C. maintained the ACE2/HEK293T cells and advised on the in vitro SARS‐CoV‐2 VSV‐GFP infection experiments. X.C, M.C., and N.A. conducted the qRT‐PCR experiments. X.C and M.N. conducted the in vivo biosafety experiment. A.T. and D.M. performed the in vivo SARS‐CoV‐2 challenge experiment. G.R. performed the mouse lung viral titers experiment. P.P.‐M. produced the SARS‐CoV‐2 VSV‐GFP pseudovirus and advised on the in vitro SARS‐CoV‐2 VSV‐GFP infection experiments. X.C. and J.H. analyzed the data and drafted the manuscript with input from M.C., A.P., N.A., J.R., and A.T., J.F., E.C., B.T., and J.H. contributed to the funding acquisition.

## Supporting information

Supporting Information

## Data Availability

Research data are not shared.
