# Supplementary material for: A Multifunctional Neutralizing Antibody‐Conjugated Nanoparticle Inhibits and Inactivates SARS‐CoV‐2
Source: Adv Sci (Weinh). 2021 Nov 10;9(2):2103240. doi: 10.1002/advs.202103240 (PMC8646742; doi:10.1002/advs.202103240)
Supplement: Supplementary file 1 — Supporting Information [file ADVS-9-0-s001.pdf]

ADVANCED SCIENCE
Open Access

Supporting Information

for *Adv. Sci.*, DOI: 10.1002/advs.202103240

A Multifunctional Neutralizing Antibody-Conjugated Nanoparticle Inhibits and Inactivates SARS-CoV-2

*Xiaolei Cai, Min Chen, Aleksander Prominski, Yiliang Lin, Nicholas Ankenbruck, Jillian Rosenberg, Mindy Nguyen, Jiuyun Shi, Anastasia Tomatsidou, Glenn Randall, Dominique Missiakas, John Fung, Eugene B. Chang, Pablo Penaloza-MacMaster, Bozhi Tian, and Jun Huang**

Supporting Information

A Multifunctional Neutralizing Antibody-Conjugated Nanoparticle Inhibits and Inactivates SARS-CoV-2

Xiaolei Cai,[1] Min Chen,[1] Aleksander Prominski,[2] Yiliang Lin,[2] Nicholas Ankenbruck,[1] Jillian Rosenberg,[3] Mindy Nguyen,[1] Jiuyun Shi,[2] Anastasia Tomatsidou,[4] Glenn Randall,[4] Dominique Missiakas,[4] John Fung,[5] Eugene B. Chang,[6] Pablo Penaloza-MacMaster,[7] Bozhi Tian,[2] and Jun Huang[1]*

[1]Pritzker School of Molecular Engineering, University of Chicago, Chicago, IL 60637, USA

[2]Department of Chemistry, University of Chicago, Chicago, IL 60637, USA

[3]Committee on Cancer Biology, University of Chicago, Chicago, IL 60637, USA

[4]Department of Microbiology, Howard Taylor Ricketts Laboratory, University of Chicago, Chicago, IL 60637, USA

[5]Department of Surgery, University of Chicago, Chicago, IL 60637, USA

[6]Department of Medicine, University of Chicago, Chicago, IL 60637, USA

[7]Department of Microbiology-Immunology, Northwestern University, Chicago, IL 60611, USA

[*]Corresponding author: huangjun@uchicago.edu

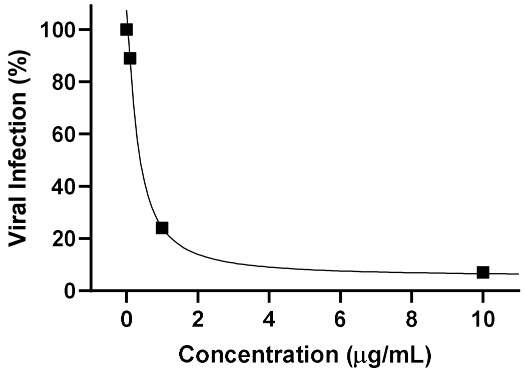

Figure S1. Neutralizing efficiency of anti-SARS-CoV-2 neutralizing antibody evaluated by the bioluminescence-based pseudotyped virus neutralization assay (provided by Sino Biological).

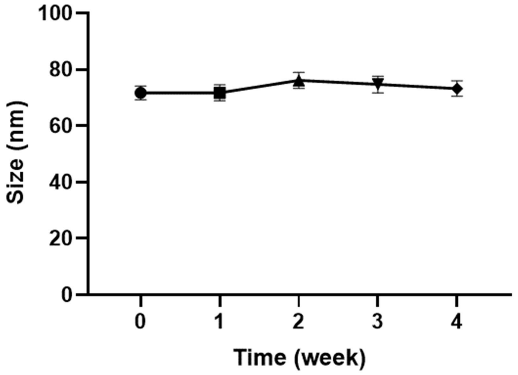

Figure S2. Hydrodynamic sizes of the multifunctional nanoparticles after storage in 1 × PBS at 4 °C over time. The hydrodynamic size of each time point was measured by DLS three times. The data are presented as mean ± SEM.

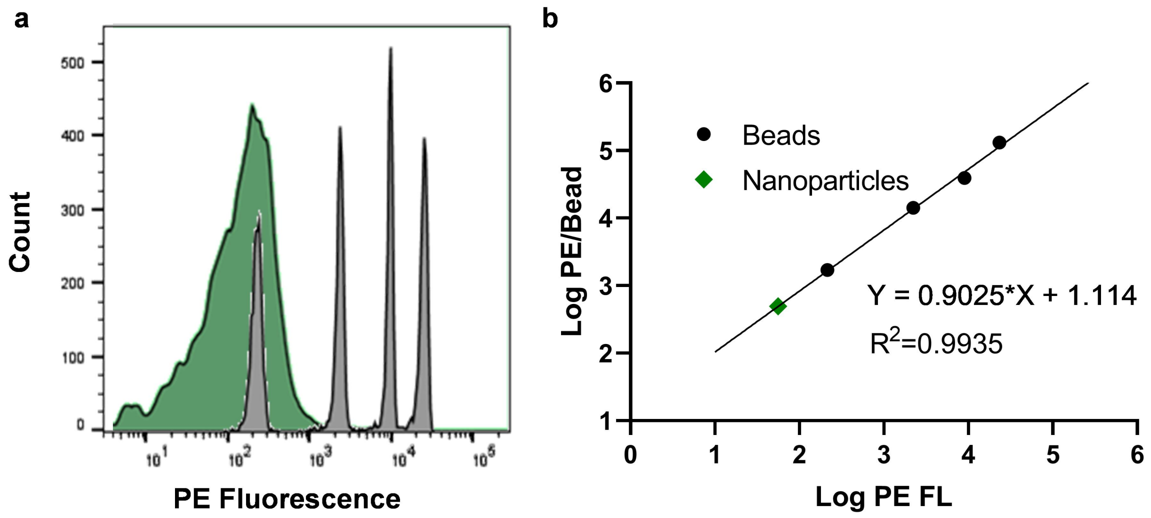

Figure S3. Quantification of surface antibody density on the multifunctional nanoparticles. **a**) Histogram displaying PE Quantibrite Beads (BD Biosciences, black) and multifunctional nanoparticles labeled with anti-IgG-PE (green). **b**) Average surface density calculated following manufacturer's instructions (BD Biosciences). The average surface densities are calculated as 1.9 $\times 10^2$ neutralizing antibodies per nanoparticle.

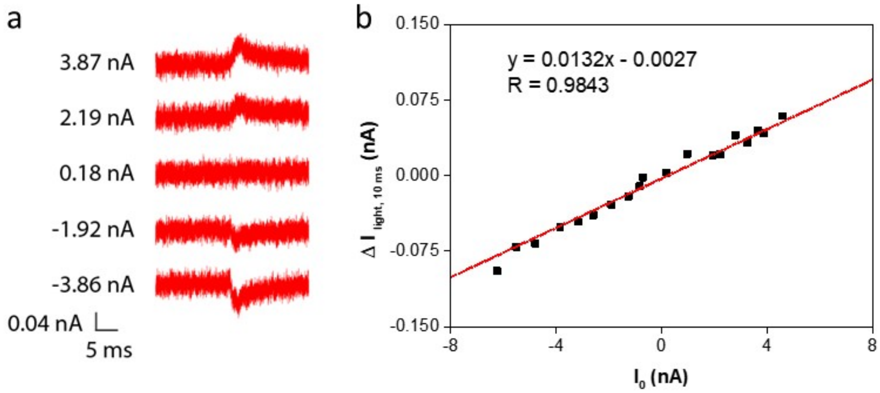

Figure S4. Photothermal evaluation using the micropipette method. **a**) Representative current traces during photoresponse experiment. **b**) Example of photoresponse curve measured at 10 ms timepoint.

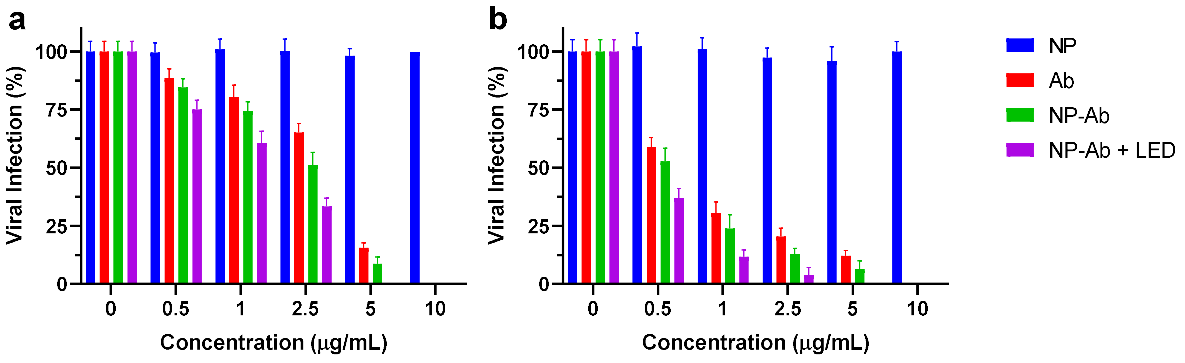

Figure S5. SARS-CoV-2 VSV-GFP pseudovirus and SARS-CoV-2 lentivirus-GFP pseudovirus infection in ACE2/HEK293T cells. **a**) Quantification of SARS-CoV-2 VSV-GFP infectivity after treatment with different concentrations of NP, Ab, NP-Ab, or NP-Ab + LED. Error bars indicate ± SEM. n = 3 per group. **b**) Quantification of SARS-CoV-2 lentivirus-GFP infectivity after treatment with different concentrations of NP, Ab, NP-Ab, or NP-Ab + LED excitation. Error bars indicate ± SEM. n = 3 per group.

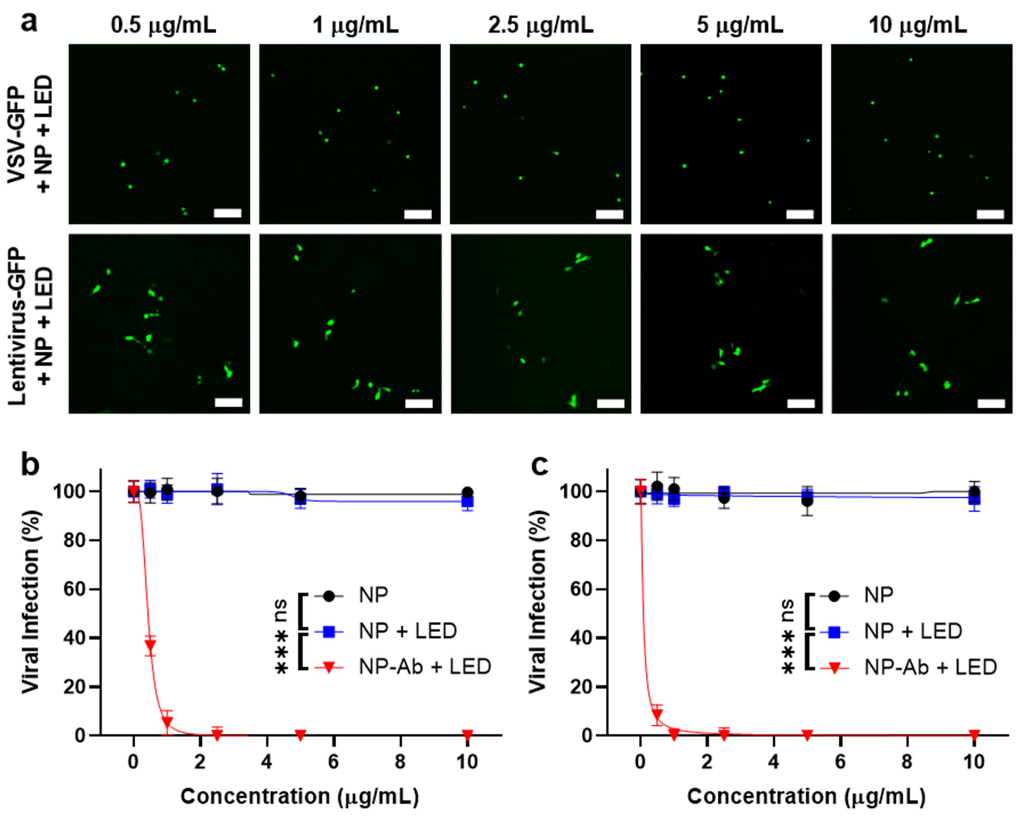

Figure S6. SARS-CoV-2 pseudoviruses infection in ACE2/HEK293T cells. **a)** Representative fluorescent images of ACE2/HEK293T cells after incubation with SARS-CoV-2 VSV-GFP or SARS-CoV-2 lentivirus-GFP treated by different concentrations of NP, NP + LED, or NP-Ab + LED. Scale bars: 100 µm. **b)** Quantification of SARS-CoV-2 VSV-GFP infectivity after treatment with different concentrations of NP, NP + LED, or NP-Ab + LED. Error bars indicate ± SEM. n = 3 per group. Statistical significance was determined by sum-of-squares F test; *** $P < 0.001$; ns: not significant, $P > 0.05$. **c)** Quantification of SARS-CoV-2 lentivirus-GFP infectivity after treatment with different concentrations of NP, NP + LED, or NP-Ab + LED. Error bars indicate ± SEM. n = 3 per group. Statistical significance was determined by sum-of-squares F test; *** $P < 0.001$; ns: not significant, $P > 0.05$.

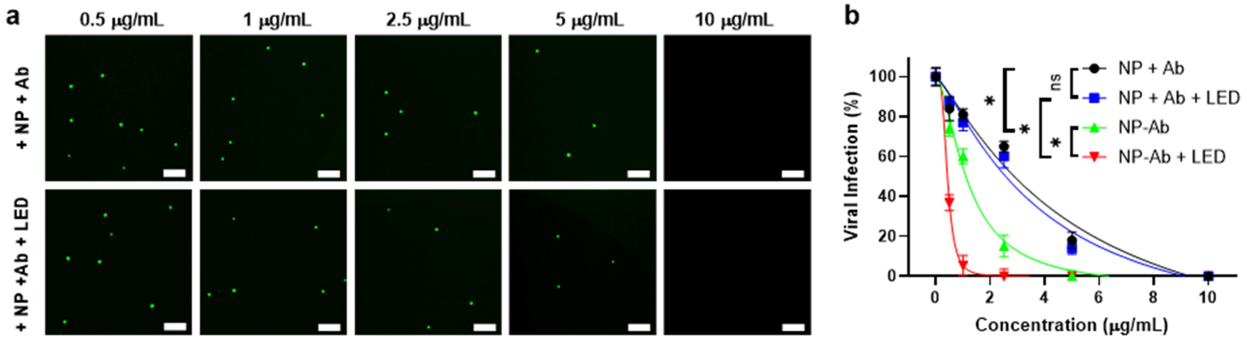

Figure S7. SARS-CoV-2 VSV-GFP pseudovirus infection in ACE2/HEK293T cells. **a**) Representative fluorescent images of ACE2/HEK293T cells after incubation with SARS-CoV-2 VSV-GFP treated by different concentrations of the mixture of NP and soluble Ab with or without 650-nm LED excitation, or NP-Ab with or without 650-nm LED excitation. Scale bars: 100 µm. **b)** Quantification of SARS-CoV-2 VSV-GFP infectivity after treatment with different concentrations of the mixture of NP and soluble Ab with or without 650-nm LED excitation, or NP-Ab with or without 650-nm LED excitation. Statistical significance was determined by sum-of-squares F test; * $P < 0.05$; ns: not significant, $P > 0.05$.

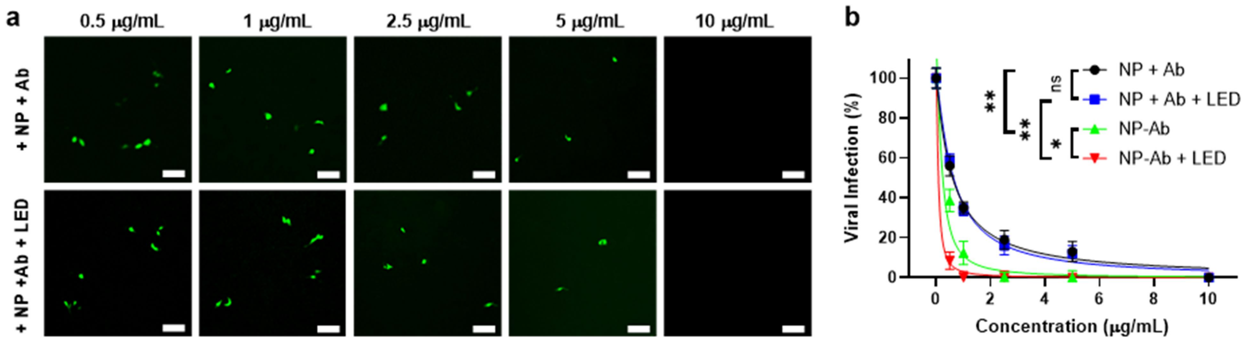

Figure S8. SARS-CoV-2 lentivirus-GFP pseudovirus infection in ACE2/HEK293T cells. **a**) Representative fluorescent images of ACE2/HEK293T cells after incubation with SARS-CoV-2 lentivirus-GFP treated by different concentrations of the mixture of NP and soluble Ab with or without 650-nm LED excitation, or NP-Ab with or without 650-nm LED excitation. Scale bars: 100 μm. **b)** Quantification of SARS-CoV-2 lentivirus-GFP infectivity after treatment with different concentrations of the mixture of NP and soluble Ab with or without 650-nm LED excitation, or NP-Ab with or without 650-nm LED excitation. Statistical significance was determined by sum-of-squares F test; * $P < 0.05$; ** $P < 0.01$; ns: not significant, $P > 0.05$.

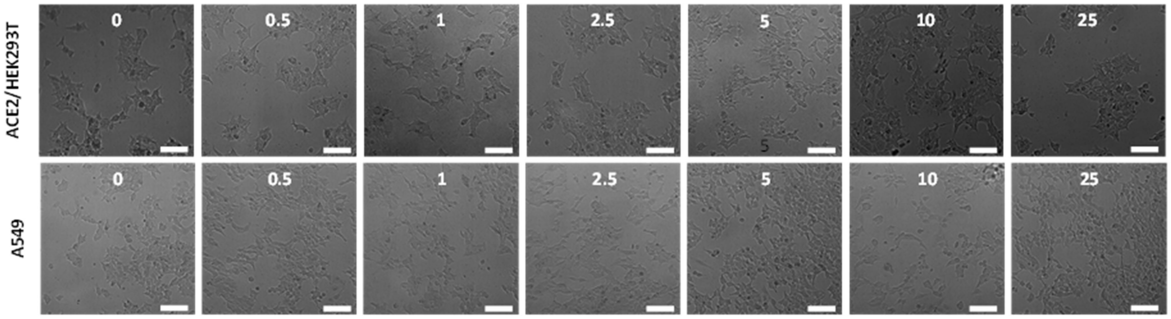

Figure S9. Bright-field images of ACE2/HEK293T cells and A549 cells after incubating with different concentrations (µg/mL) of the multifunctional nanoparticles. Scale bars: 50 µm.

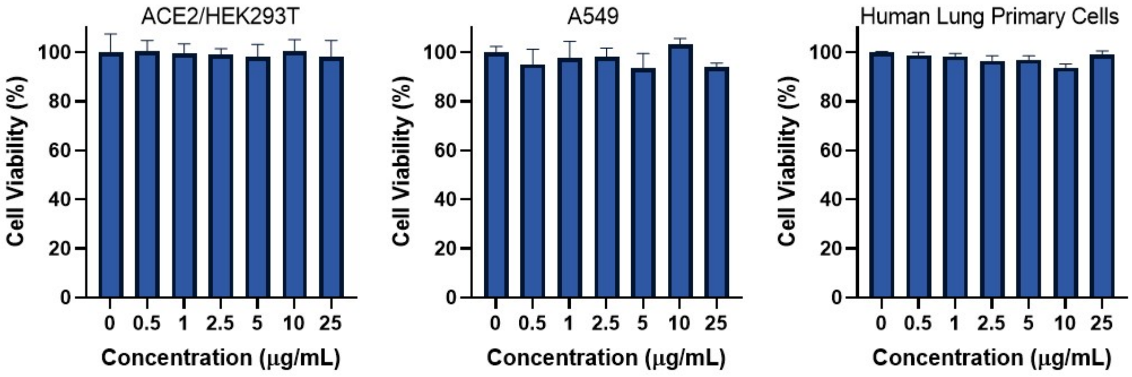

Figure S10. Cell viabilities of ACE2/HEK293T, A549, and human lung primary cells after incubation with different concentrations of the multifunctional nanoparticles. Error bars indicate ± SEM. n = 3 per group.

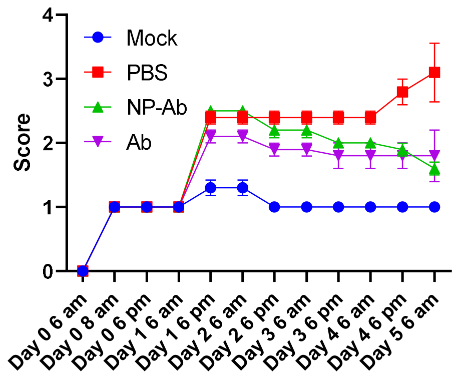

Figure S11. Mouse clinical scoring after SARS-CoV-2 challenge under different treatments. Error bars indicate ± SEM. n = 5 per group. Score 0 (pre-inoculation) - animal is bright, alert, active, with normal fur coat and posture; Score 1 (post-inoculation, pi) – animal is bright, alert, active, normal fur coat and posture, no weight loss; Score 1.5 - animal has slightly ruffled fur but is active; weight loss under 2.5%; Score 2 (pi) – animal has ruffled fur, is less active; weight loss under 5%; Score 2.5 (pi) - animal has ruffled fur, is not active but moves when touched, may have hunched posture or difficulty breathing; weight loss 5-10%; Score 3 (pi) – same as score 2.5; weight loss 11- 20%; Score 4 (pi) - animal has ruffled fur or is positioned on its side or back, dehydrated, has difficulty breathing; weight loss >20%; Score 5 (pi) – death.

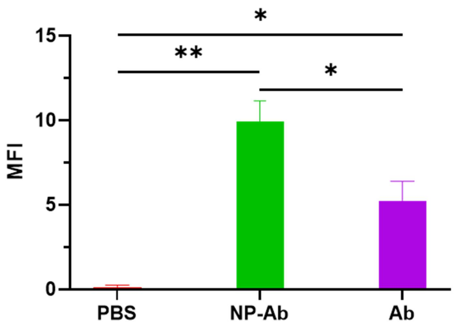

Figure S12. Mean fluorescence intensity of the anti-IgG-PE in immunostained mouse lung sections after intranasal injection of PBS, NP-Ab, or Ab. Error bars indicate ± SEM. n = 5 per group. Statistical significance was determined by two-tailed unpaired t-test; * $P < 0.05$; ** $P < 0.01$.

Experimental Section

Materials

Poly[2,6-(4,4-bis-(2-ethylhexyl)-4H-cyclopenta [2,1-b;3,4-b]dithiophene)-alt-4,7(2,1,3-benzothiadiazole)] (PCPDTBT) and tetrahydrofuran (THF) were purchased from Sigma-Aldrich. 32% paraformaldehyde aqueous solution was purchased from Electron Microscopy Sciences. 1,2-Distearoyl-sn-glycero-3-phosphoethanolamine-N-[carboxy(polyethylene glycol)-2000, NHS ester] (DSPE-PEG_{2000}-NHS) was ordered from Avanti Polar Lipids. 1,1'-Dioctadecyl-3,3,3',3'-tetramethylindotricarbocyanine iodide) (DiR) was purchased from Biotium. Monoclonal anti-SARS-CoV-2 neutralizing antibody (Mouse IgG2b, Clone #57) was purchased from Sino Biological. Alexa Fluor 488-labeled anti-mouse IgG2b antibody (clone RMG2b-1) was purchased from BioLegend. CellTiter-Glo® Luminescent cell viability assay was purchased from Promega. SARS-CoV-2 pseudotyped lentivirus with GFP reporter gene and luciferase reporter gene were purchased from Integral Molecular. Anti-IgG-PE was purchased from BioLegend. Anti-IgG2b-HRP was purchased from Abcam. ACE2/HEK293T cells were provided by Integral Molecular. Human lung epithelial cell line A549 and Vero E6 cell line were obtained from ATCC®. All the cells were cultured in Dulbecco's Modified Eagle Medium (DMEM) with High Glucose and L-glutamine. Media was supplemented with 10% fetal bovine serum (FBS; Thermo Fisher Scientific) and 1% penicillin-streptomycin (PS; Life Technologies) and cells were maintained at 37 °C in 5% CO_2 atmosphere.

Preparation of the multifunctional nanoparticles

PCPDTBT (0.2 mg) and DSPE-PEG_{2000}-NHS (2 mg) were dissolved and mixed in THF (500 µL).[47] For DiR labeled nanoparticles, 0.04 mg of DiR was further added into the mixture. The mixture was injected into 1 × PBS (10 mL) and sonicated using a probe sonicator (Qsonica Ultrasonic Homogenizer) on ice for 2 min. THF was then evaporated by rotary evaporator. The obtained nanoparticles were filtered through a 0.22 µm syringe filter and concentrated by ultrafiltration (Amicon® Ultra-15 Centrifugal Filter Unit with 100 KDa cutoff, 4000 × g, 4 °C, 5 min). Anti-SARS-CoV-2 neutralizing antibodies were added into the nanoparticle solution with a mass ratio of 1:10 (antibody: DSPE-PEG_{2000}-NHS) and stirred overnight at 4 °C to complete the conjugation.

Nanoparticle characterization

The measurement of UV-vis absorption spectra was carried out using a UV-vis spectrophotometer (Thermo Scientific NanoDrop™ 2000). The sizes of the nanoparticles were measured by dynamic light scattering (DLS) particle size analyzer (Malvern Zetasizer). The sizes and morphologies of the nanoparticles were studied by transmission electron microscopy (TEM, JEM-2010F, JEOL, Japan).

The surface neutralizing antibody conjugation was firstly investigated by fluorescent imaging of the multifunctional nanoparticles. The multifunctional nanoparticles (100 μg/mL, 10 μL) was incubated with the secondary Alexa Fluor 488-labeled anti-IgG2b antibody (100 μg/mL, 10 μL) for 1 h. Single particle fluorescent images were captured by a fluorescence microscopy with a 100×/1.49 numerical aperture (NA) objective using a Nikon Ti-E inverted microscope. A 7-color solid state LED light source was connected with a liquid light guide to the microscope; the light then passes through a quad band-pass filter (ZET405-488-532-647m). The LED excitation wavelengths used were 470 ± 25 nm for green channel and 740 ± 20 nm for red channel (Spectra X, Lumencor). The emissions from the nanoparticles and Alexa Fluor-488 were captured by an Andor iXon Ultra 888 back-illuminated electron multiplying CCD (EMCCD) camera (Oxford Instruments).

The density of the neutralizing antibody on the multifunctional nanoparticle surface was measured by flow cytometry.[1] DiR-loaded nanoparticles was stained with anti-IgG-PE (BioLegend®) for 30 min on ice. DiR^+PE^+ double-positive populations were compared with PE Quantitation Beads (BD Quantibrite) to calculate surface antibody densities according to the manufacturer's instructions (Figure S3). The total antibody conjugation efficiency was calculated to be ~ 22% by the follow equation:

$$\textbf{Antibody conjugation efficiency} = \frac{\frac{\text{Ab surface density} * \text{Nanoparticles density} * \text{V}}{\text{N}} * \text{MAb}}{\text{Total antibody} * \text{V}} = \frac{\text{Ab surface density} * \text{Nanoparticles density} * \text{MAb}}{\text{Total antibody} * \text{N}}$$

Ab surface density: number of antibodies on the surface of one single nanoparticle; Nanoparticle density (mL^{-1}): number of nanoparticles in the multifunctional nanoparticle solution counted by microscope; V (mL): volume of the multifunctional nanoparticle solution; MAb (g/mol): molecular weight of antibody = 150000 g/mol; N (mol^{-1}): Avogadro Number = 6.02×10^{23} mol^{-1}; Total antibody (g/mL): total antibody concentration in the multifunctional nanoparticle solution.

Photothermal test of the multifunctional nanoparticles

Multifunctional nanoparticles (100 μg/mL in 1 × PBS) were added into a well in a 96-well plate (flat bottom, GenClone®). A 650-nm LED (Spectra X, Lumencor) was applied to excite the nanoparticle solution for 10 min with a power density of 250 mW/cm^2. The temperature changes from 0 to 10 min were recorded by a probe thermometer. Each measurement was repeated three times. Temperature changes of pure 1 × PBS were also measured using the same method.

Measurement of transient photothermal response of multifunctional nanoparticles

Photothermal response measurements were performed on an upright microscope (Olympus, BX61WI) with a 20×/0.5 NA water-immersion objective. An LED light source (Lumencore Spectra X) with a 650 nm filter was used for excitation and was electronically controlled using transistor-transistor logic signals delivered from a digitizer (Molecular Devices, Digidata 1550). Voltage-clamp measurements were performed using Axopatch 200B amplifier (Molecular Devices). For the local temperature measurements, a pipette electrode (~0.8 MΩ at room temperature) filled with buffer solution (1 × PBS) was placed in a close proximity to the nanoparticles aggregated on top of glass coverslip submerged in PBS solution. Heat-induced currents were recorded in voltage-clamp mode. A laser pulse of 10 ms was delivered to the preparation 100 ms after the voltage jumped 0.5 mV below the holding potential. Current traces were recorded for the set of holding currents (Figure S4a) and the linear relationship between the holding current and photothermal current was established for different time points (Figure S4b). Temperature was inferred using a temperature-resistance calibration curve measured for the pipette.

ELISA testing of the antibody binding affinity

ELISA plate (Biolegend®) was incubated with 100 μl anti-SARS-CoV-2 neutralizing antibodies (1 μg/mL in 1 × Coating Buffer, Biolegend®) overnight at 4 °C. The plate was washed four times with 300 μl of PBS + 0.05% Tween-20 and incubated with 200 μl of 1 × Assay Diluent (Biolegend®) at room temperature for 1 h with shaking. The plate was washed four times with 300 μl of PBS + 0.05% Tween-20 and incubated with 100 μl Biotinylated SARS-CoV-2 spike protein (1 μg/mL in 1 × Assay Diluent) at room temperature for 1 h with shaking. The plate was washed four times with 300 μl of PBS + 0.05% Tween-20 and heated by hot plates to 37 °C,

50 °C or 65 °C for 10 mins. The plate was washed four times with 300 μl of PBS + 0.05% Tween-20 and incubated with 100 μL of Avidin-HRP solution (Biolegend®) at room temperature for 30 min with shaking. Then the plate was washed five times with 300 μl of PBS + 0.05% Tween-20 and incubated with 100 μL freshly mixed TMB Substrate Solution (Biolegend®) and incubate in the dark for 30 minutes. 100 μL 2N H_2SO_4 was added into each well to stop the reaction. The absorbance at 450 nm was read by a microplate reader (Fisher Scientific BioTek Cytation 5).

SARS-CoV-2 pseudotyped VSV production

SARS-CoV-2 VSV-GFP was produced using VSVdG*G carrying the spike protein from the Wuhan-Hu-1 strain of SARS-CoV-2 (GenBank: MN908947) as described previously.[2] Briefly, HEK293T cells were seeded at a density of 70% in a T-125 flask in DMEM supplemented with 10% FBS. The following day, media was replaced with FBS-free DMEM and cells were transfected with pCAGGS-SARS-CoV-2 S protein (BEI; cat#: NR-52310) using polyethylenimine (PEI). After 24 h, VSVdG*G was added at an MOI of 3. After 2 h, media was supplemented with DMEM containing 10% FBS. After 48 h, the SARS-CoV-2 VSV-GFP particles were harvested and further purified via size exclusion column.

***In vitro* viral infection of SARS-CoV-2 VSV-GFP pseudovirus**

ACE2/HEK293T cells (5×10^4 cells) were seeded into 96-well plates (flat bottom, GenClone®) and incubated overnight at 37 °C in a humified CO_2 incubator. A dilution series of the NP, Ab, and NP-Ab were prepared in a final volume of 50 μL of Dulbecco's Modified Eagle Medium (DMEM) for a single well. Simultaneously, 15 μL of SARS-CoV-2 VSV-GFP (2×10^5 titering units (TU)/mL) was diluted into DMEM to a final volume of 50 μL for a single well and combined with the NP, Ab, NP-Ab, or a mixture of NP and Ab to a total volume of 100 μL and incubated at 37°C for 1 h. After the initial incubation, one group of the mixtures containing NP-Ab were excited by the 650-nm LED for 10 min with a power density of 250 mW/cm^2. All the mixtures were added into the cells. After 2 h incubation, 100 μL of fresh cell culture media was further added into each well. Each group contains three wells for the subsequent imaging and quantification. After 24 h incubation, the cells were imaged with Leica fluorescence microscope using 10×/0.30 NA objective. The LED excitation wavelengths were 470 ± 25 nm for GFP (Spectra X, Lumencor). The emissions were captured by an Andor iXon Ultra 888 back-

illuminated EMCCD camera (Oxford Instruments). GFP-positive cells were counted manually three times by objective and the viral infection (%) were calculated as the ratio of GFP-positive cells in the group incubated with the nanoparticles and virus to that of the group incubated with virus alone.

***In vitro* viral infection of SARS-CoV-2 lentivirus-GFP pseudovirus**

ACE2/HEK293T cells (2×10^4 cells) were seeded into 96-well plates (flat bottom, GenClone®) and incubated overnight at 37 °C in a humified CO_2 incubator. A dilution series of the NP, Ab, and NP-Ab were prepared in a final volume of 50 µL of cell culture media for a single well. Simultaneously, 10 µL of SARS-CoV-2 lentivirus-GFP (1×10^6 TU/mL) was diluted into cell culture media to a final volume of 50 µL for a single well and combined with the NP, Ab, NP-Ab, or a mixture of NP and Ab to a total volume of 100 µL and incubated at 37°C for 1 h. After the initial incubation, one group of the mixtures containing NP-Ab were excited by the 650-nm LED for 10 min with a power density of 250 mW/cm^2. All the mixtures were added onto the cells. After 48 h incubation, cells were washed with 1 × PBS and fixed in 4% [w/v] paraformaldehyde for 15 min. Cells were then washed with 1 × PBS and further stained with Hoechst 33342 nuclear stain (2 µg/mL) for 15 min. The GFP expression monitored with a Nikon Ti-E inverted microscope using a 10×/0.30 NA objective. The LED excitation wavelengths were 395 ± 25 nm for Hoechst 33342 and 470 ± 25 nm for GFP (Spectra X, Lumencor). The emissions were captured by an Andor iXon Ultra 888 back-illuminated EMCCD camera (Oxford Instruments). The numbers of total cells and infected cells were counted in ImageJ using the following workflow: Adjust→Threshold (Otsu), Process→Binary→Fill Holes→Watershed, Analyze→Analyze Particles. GFP expression was first normalized to total number of cells as calculated by DAPI staining. Then, viral infection (%) were calculated as the ratio of normalized GFP positive cells in the group incubated with the virus and nanoparticles to that of the group incubated with virus only.

***In vitro* viral inactivation efficiency calculation**

The viral infection (%) of SARS-CoV-2 VSV-GFP and SARS-CoV-2 lentivirus-GFP in ACE2/HEK293T cells after NP, Ab, NP-Ab or NP-Ab + LED treatments were quantified as illustrated above (Figure S5). Since the surface antibody conjugation efficiency of the multifunctional nanoparticles is 22%, the nanoparticle solution containing free antibodies. By

assuming that the free antibodies in the nanoparticle solution have the same virus inactivation function as the pure antibodies, the actual viral inactivation efficiency of the multifunctional nanoparticle was calculated by the following equations:

$$\text{Viral inactivation efficiency of Pure Ab} = \frac{\text{Total viral particles} * (1 - \text{Percentage of viral infection after pure Ab treatment})}{\text{Total Pure Ab}} \quad (1)$$

$$\text{Viral inactivation efficiency of NP} - \text{Ab} = \frac{\text{Total viral particles} - \text{Actual free ab} * \text{Inactivation efficiency of Pure Ab}}{\text{Actual NP} - \text{Ab}} \quad (2)$$

By combining equation (1) and (2), the actual viral infection (%) was calculated:

$$\text{Actual viral infection (\%) after NP} - \text{Ab treatment} = 1 - \frac{\text{Total NP} - \text{Ab} * \text{Inactivation efficiency of NP} - \text{Ab}}{\text{Total viral particles}}$$

$$= 1 - \frac{\text{Total NP} - \text{Ab} * \text{Total viral particles} * \frac{\left(1 - \frac{(1 - \text{Percentage of viral infection after pure Ab treatment}) * \text{Actual free Ab}}{\text{Total Pure Ab}}\right)}{\text{Actual NP} - \text{Ab}}}{\text{Total vrial particles}}$$

$$== 1 - \text{Total NP} - \text{Ab} * \frac{\left(1 - \frac{(1 - \text{Percentage of viral infection after pure Ab treatment}) * \text{Actual free Ab}}{\text{Total Pure Ab}}\right)}{\text{Actual NP} - \text{Ab}}$$

qRT-PCR quantification of viral infection in ACE2/HEK293T cells

ACE2/HEK293T cells (1×10^6 cells) were seeded in a 6-well plate (CELLTREAT®). The first group of cells were not treated and served as the control. The second group of cells were incubated with 20 ul SARS-CoV2 pseudotyped lentivirus with luciferase reporter gene (SARS-CoV-2 lentivirus-luciferase). The third group of cells were incubated with 20 ul SARS-CoV2 pseudotyped lentivirus treated with 5 ug/ml NP-Ab and irradiated by a 650-nm LED for 10 min. Cells were harvested after 48 h incubation with virus, cell pellets were used for RNA extraction (RNAeasy Mini Kit, Qiagen) according to the manufacturer's instructions. RNA was quantified using a Nanodrop-2000. Complementary DNA (cDNA) was synthesized from DNAse-free RNA (1 µg) using oligo(dT) and random primers and the GoScript™ Reverse Transcription System kit (Promega) according to the manufacturer's instructions. The resulting cDNA was analyzed in triplicate for GAPDH and *Renilla* luciferase expression using the Applied Biosystems PowerUp SYBR Green Master Mix (Thermo Fisher Scientific) with commercial primers and the following program: i) 50 °C for 2 minutes, ii) 95 °C for 2 minutes, and iii) 40 cycles of 95 °C for 15

seconds, 50 °C for 15 seconds and 1 minute at 72 °C. Fold changes were determined using the $2^{-\Delta\Delta Ct}$ method[3] by comparing treated groups with the control group. The mRNA expression of *Renilla* luciferase was normalized to GAPDH expression for each sample. Primers used for RT-qPCR are listed below: GADPH forward primer (5'-GAAGGTGAAGGTCGGAGTC-3'), GADPH reverse primer (5'-GAAGATGGTGATGGGATTTC-3'), *Renilla* luciferase forward primer (5'-AATTTGCAGCATATCTTGAACCATT-3'), *Renilla* luciferase reverse primer (5'-GGATTTCACGAGGCCATGAT-3').

***In vitro* viral infection of SARS-CoV-2 lentivirus-luciferase pseudovirus**

Vero E6 cells and human lung primary cells (1×10^4 cells) were seeded into 384-well plates (flat bottom, UltraCruz®). NP, Ab, or NP-Ab were mixed with 4 µL of SARS-CoV-2 lentivirus-luciferase (1×10^6 TU/mL) in cell culture media and incubated at 37°C for 1 h. After the initial incubation, one group of the mixtures containing NP-Ab were further excited by the 650-nm LED for 10 min with a power density of 250 mW/cm^2. All the mixtures were added into the cells (n = 5 per group). After 48 h incubation, the plates were centrifuged for 5 min at 500 × g to prevent cell loss. Supernatant was aspirated and 35 µL of PBS was added. PBS was carefully aspirated, leaving ~15 µL of liquid behind. 15 µL of *Renilla*-Glo Assay Substrate (1:100 dilution) was added to each well. Bioluminescence was recorded by a microplate reader (Fisher Scientific BioTek Cytation 5) with an exposure of 200 ms. Wells infected with pseudovirus only were normalized as 100%.

***In vitro* cytotoxicity test**

ACE2/HEK293T cells, A549 cells and human lung primary cells were seeded into 96-well plates (flat bottom, GenClone®) and incubated with a dilution series of the multifunctional nanoparticles for 24 h. The morphologies of the ACE2/HEK293T cells and A549 cells were monitored with a Nikon Ti-E inverted microscope using a 10×/0.30 NA objective. The cell viability was determined by adding 100 µL of freshly prepared CellTiter-Glo® reagent into each well. The plate was gently shaken for 2 minutes and further incubated for 10 min at room temperature. Luminescence was measured by a microplate reader (Fisher Scientific BioTek Cytation 5). The luminescent signal for cells treated with the multifunctional nanoparticles was normalized to that of the cells incubated with culture medium only.

***In vivo* biosafety test**

C57BL/6NHsd mice at the age of 6 weeks were purchased from Envigo and maintained at the Animal Facility of the University of Chicago. The animal study protocols were approved by the Institutional Animal Care and Use Committee of the University of Chicago. For evaluation of the safety of the multifunctional nanoparticles, two male and two female 6- to 10-week-old C57BL/6NHsd mice were intratracheally administered with 50 μL of the multifunctional nanoparticle solutions (10 mg/kg). At day-3 post administration, blood samples were collected by submandibular vein via cheek punch using a commercially available 4-mm point lancet. A small aliquot of approximately 100 μL of blood was collected into EDTA-containing heparinized tubes, and RBCs, WBCs, and platelets were counted. For the comprehensive chemistry panels, blood was allowed to coagulate at 4°C for 2 h, and serum was collected after centrifugation (1,000 × *g* for 15 min) for analysis. Serum alkaline phosphatase, alanine aminotransferase, amylase, urea nitrogen, calcium, cholesterol, glucose, total bilirubin, and total proteins were determined by a Vet Axcel blood chemistry analyzer (Alfa Wasserman). Lungs, heart, liver, spleen, and kidney were collected, fixed in 10% formalin for 24 h, and embedded in paraffin. The resulting blocks were cut into 5-µm sections and further stained with H&E by the University of Chicago Human Tissue Research Center. The slides were scanned by a CRi Pannoramic MIDI 20× whole-slide scanner. Fluorescent imaging samples were collected in Tissue-Tek OCT Compound on dried ice. The samples were further cryosectioned and stained with DAPI by the University of Chicago Human Tissue Research Center and imaged with a Nikon Ti-E inverted microscope using a 10×/0.30 NA objective.

***In vivo* SARS-CoV-2 treatment**

Experiments with SARS-CoV-2 were performed in biosafety level 3 (BSL3) and animal BSL3 (ABSL3) containment in accordance with the institutional guidelines following experimental protocol review and approval by the Institutional Biosafety Committee (IBC) and the Institutional Animal Care and Use Committee (IACUC) at the University of Chicago.

6-8 weeks old male B6.Cg-Tg(K18-ACE2)2Prlmn/J (K18-hACE2) mice (Jackson Laboratory) were anesthetized by intraperitoneal injection with ketamine–xylazine (100 mg–20 mg/kg), for intranasal administration of nanoparticles and virus. Animals were challenged with 2×10^4 PFU of USA-WA1/2020 SARS-CoV-2 (2019-nCoV) in 20 μL. Two hours post challenge animals

were treated with 30 uL of either PBS, multifunctional nanoparticles (10 mg/kg) or antibody (10 mg/kg). Mice were monitored twice daily to record clinical symptoms and weighed daily for 5 days post-challenge with virus. Categories in clinical scoring included: Score 0 (pre-inoculation) - animal is bright, alert, active, with normal fur coat and posture; Score 1 (post-inoculation, pi) – animal is bright, alert, active, normal fur coat and posture, no weight loss; Score 1.5 - animal has slightly ruffled fur but is active; weight loss under 2.5%; Score 2 (pi) – animal has ruffled fur, is less active; weight loss under 5%; Score 2.5 (pi) - animal has ruffled fur, is not active but moves when touched, may have hunched posture or difficulty breathing; weight loss 5-10%; Score 3 (pi) – same as score 2.5; weight loss 11- 20%; Score 4 (pi) - animal has ruffled fur or is positioned on its side or back, dehydrated, has difficulty breathing; weight loss >20%; Score 5 (pi) – death. At day five post-challenge, all animals were euthanized and subjected to necropsy to remove the lungs, heart, liver, spleen and kidney. Right lungs were homogenized for measurement of viral titers, RNA extraction and ELISA quantification. Left lungs were fixed in 10% formalin and embedded in paraffin. The resulting blocks were cut into 5-μm sections and further stained with DAPI and anti-IgG-PE (BioLegend®) by the University of Chicago Human Tissue Research Center. The slides were scanned by an Olympus VS200 slide scanner. The mean fluorescence intensity (MFI) of the PE channel of the images were measured by ImageJ.

RNA extraction from lung homogenates was performed using the RNAeasy Mini Kit (Qiagen) according to the manufacturer's instructions. RNA was quantified using a Nanodrop-2000 spectrophotometer. Complementary DNA (cDNA) was synthesized from DNAse-free RNA (500 ng) using GoScript™ Reverse Transcription System kit (Promega) according to the manufacturer's instructions. Then, GAPDH and SARS-CoV-2 (2019-nCoV_N1) expression were determined using the Applied Biosystems PowerUp SYBR Green Master Mix (Thermo Fisher Scientific) with commercial primers and the following program: i) 50 °C for 2 minutes, ii) 95 °C for 2 minutes, and iii) 40 cycles of 95 °C for 15 seconds, 50 °C for 15 seconds and 1 minute at 72 °C. Fold changes were determined using the $2^{-\Delta\Delta Ct}$ method[3] by comparing treated groups with the control group. The mRNA expression of 2019-nCoV_N1 was normalized to GAPDH expression for each sample. Primers used for RT-qPCR are listed below: GADPH forward primer (5'- TGCACCACCAACTGCTTAGC-3'), GADPH reverse primer (5'-TGCACCACCAACTGCTTAGC-3'), 2019-nCoV_N1 forward primer (5'-

GACCCCAAAATCAGCGAAAT-3’), 2019-nCoV_N1 reverse primer (5’-TCTGGTTACTGCCAGTTGAATCTG -3’). The viral titers (PFU/mL) were determined by adding the lung homogenates on a monolayer of Vero E6 cells. After three days, plaques were visualized via a plaque assay.

Lung homogenates were lysed by RIPA Lysis Buffer (Thermo Fisher). ELISA plate (Biolegend®) was incubated with 100 µl anti-SARS-CoV-2 spike proteins (1 µg/mL in 1 × Coating Buffer, Biolegend®) overnight at 4 °C. The plate was washed four times with 300 µl of PBS + 0.05% Tween-20 and incubated with 200 µl of 1 × Assay Diluent (Biolegend®) at room temperature for 1 h with shaking. The plate was washed four times with 300 µl of PBS + 0.05% Tween-20 and incubated with 100 µl of the diluted lung lysates or different concentrations of the SARS-CoV-2 antibody at room temperature for 2 h with shaking. The plate was washed four times with 300 µl of PBS + 0.05% Tween-20 and incubated with 100 µL of diluted (1:1000) anti-IgG2b-HRP solution (Abcam) at room temperature for 1 h with shaking. Then the plate was washed five times with 300 µl of PBS + 0.05% Tween-20 and incubated with 100 µL freshly mixed TMB Substrate Solution (Biolegend®) and incubate in the dark for 30 minutes. 100 µL 2N H_2SO_4 was added into each well to stop the reaction. The absorbance at 450 nm was read by a microplate reader (Fisher Scientific BioTek Cytation 5). The Ab levels were calculated by fitting the 450-nm absorbance of lung lysates with those of the SARS-CoV-2 antibody.

Statistical Analysis

The *in vitro* and *in vivo* experimental results were expressed as means ± SEM. The sample sizes are indicated in the corresponding figure legends. Statistical significance was determined as described in the corresponding figure legends. $P > 0.05$ were considered to be no significant (ns). $P < 0.05$ was considered to be statistically significant (* $P < 0.05$; ** $P < 0.01$; *** $P < 0.001$.). GraphPad Prism software was applied for data analysis.

References

1. Huang, J.; Zarnitsyna, V. I.; Liu, B.; Edwards, L. J.; Jiang, N.; Evavold, B. D.; Zhu, C. The kinetics of two-dimensional TCR and pMHC interactions determine T-cell responsiveness. *Nature* **2010,** *464*, 932-936.

2. Whitt, M. A. Generation of VSV pseudotypes using recombinant ΔG-VSV for studies on virus entry, identification of entry inhibitors, and immune responses to vaccines. *J Virol Methods* **2010,** *169*, 365-74.
3. Livak, K. J.; Schmittgen, T. D. Analysis of relative gene expression data using real-time quantitative PCR and the 2(-Delta Delta C(T)) Method. *Methods (San Diego, Calif.)* **2001,** *25*, 402-8.
